# Supplementary material for: Testicular Transcriptome of Males and Pseudo-Males Provides Important New Insight into Sex Reversal of Rana dybowskii
Source: Animals (Basel). 2022 Oct 21;12(20):2887. doi: 10.3390/ani12202887 (PMC9598223; doi:10.3390/ani12202887)
Supplement: Supplementary file 1 [file animals-12-02887-s001.zip › Supplementary Table 1.pdf]

Table S1

Primers used for quantitative real-time PCR

Gene	Primers (5′-3′)	Size (bp)	TM (°C)
β-actin	F: GGATCAGCAAGCAGGAGTA<br>R: GAAAGCCATGCCAGTGAT	117	54.0
Dmrt1	F:TAAGCAAGCCTCGCAAACCT<br>R:ACTGACATTCCCGCCACATA	150	60.3
3β-HSD	F:GTATCACCGACGCCATCCAATCAC<br>R:CACCTGCTGACCATCCTGAACAC	133	65.0
CYP26B1	F:CCTCCTCCTCCTCCTCCTCCTC<br>R:AGCACCGACACCAGATCAAAGC	92	64.0

F, forward primer; R, reverse primer. *Dmrt1*, doublesex and mab-3 related transcription factor 1; *3 β -HSD*, 3-beta-hydroxysteroid dehydrogenase; *CYP26B1*, cytochrome P450 26B1.
